# Development and validation of cuproptosis-associated prognostic signatures in WHO 2/3 glioma

**DOI:** 10.3389/fonc.2022.967159

**Published:** 2022-08-18

**Authors:** Zhang Ye, Shenqi Zhang, Jiayang Cai, Liguo Ye, Lun Gao, Yixuan Wang, Shiao Tong, Qian Sun, Yu Wu, Xiaoxing Xiong, Qianxue Chen

**Affiliations:** ^1^ Department of Neurosurgery, Renmin Hospital of Wuhan University, Wuhan, China; ^2^ Central Laboratory, Renmin Hospital of Wuhan University, Wuhan, China

**Keywords:** cuproptosis-associated risk signatures, prognosis, FDX1, CDKN2A, WHO 2/3 glioma

## Abstract

WHO 2/3 glioma is a common intracranial tumor that seriously affects the quality of life and survival time of patients. Previous studies have shown that the tricarboxylic acid (TCA) cycle is closely related to the occurrence and development of glioma, while recent studies have shown that cuproptosis, a novel programmed death pathway, is closely related to the inhibition of the TCA cycle. In our study, eight of ten cuproptosis-related genes (CRGs) were found to be differentially expressed between normal and WHO 2/3 glioma tissues. Through the LASSO algorithm, the cuproptosis-associated risk signatures (CARSs) were constructed, which can effectively predict the prognosis of WHO 2/3 glioma patients and are closely related to clinicopathological features. We analyzed the relationship between risk score and immune cell infiltration through Xcell, ssGSEA, TIMER database, and immune checkpoint molecules. In addition, the relationship between risk score and chemotherapeutic drug sensitivity was also investigated. The prognosis-related independent risk factors FDX1 and CDKN2A identified from CARSs are considered potential prognostic biomarkers for WHO 2/3 glioma. The clinical prognosis model based on cuproptosis is expected to provide an effective reference for the diagnosis and treatment of clinical WHO 2/3 glioma patients.

## Introduction

Glioma is the most common primary malignant brain tumor. They are classified into WHO 1-4 gliomas according to their pathological features. Patients with WHO 2/3 glioma have a better prognosis than those with glioblastoma multiforme. However, many treatments, especially radiation therapy, produce or cause chronic damage (1). Surgical resection and chemotherapy are still the main treatments for WHO 2/3 glioma (2). However, due to the lack of obvious clinical symptoms, most patients miss the best time for surgical treatment. Temozolomide, as a first-line chemotherapy strategy, also has the risk of inducing acquired resistance (3). Therefore, the identification of novel biomarkers in WHO 2/3 glioma patients is crucial for the treatment of WHO 2/3 glioma.

Copper participates in various biological metabolic processes in the human body. Recently, Tsvetkov et al. discovered a novel cell death pathway based on intracellular copper overload, termed cuproptosis (4). Excessive intracellular accumulation of copper binds directly to fatty acylated components of the tricarboxylic acid (TCA) cycle, and then aggregation of these copper-bound fatty acylated mitochondrial proteins and subsequent loss of Fe-S cluster proteins trigger proteotoxic stress and cuproptosis (5). Glioma is closely associated with TCA cycle reprogramming, enabling tumor cells to survive nutrient depletion and hypoxia (6).

In our study, we speculate that cuproptosis-related genes (CRGs) have special prognostic significance for WHO 2/3 glioma. Novel CARSs developed and validated through various bioinformatic approaches may be potentially incorporated into existing clinicopathological characterization and staging systems to improve outcomes for WHO 2/3 glioma patients. Moreover, we also found that CARS can provide evidence for immunotherapy and chemotherapy in WHO 2/3 glioma patients.

## Materials and methods

### Public data and sample collection

We screened 529 WHO 2/3 glioma samples from The Cancer Genome Atlas (TCGA) and collected RNA-seq data as well as clinical information as a training cohort; similarly, we screened 159 WHO 2/3 glioma samples from the Chinese Glioma Genome Atlas (CGGA) and collected RNA-seq data as well as clinical information as a validation cohort. In this study, cases with survival ≤30 days or no survival data were excluded, while cases with complete mRNA expression data and corresponding clinical information were used for subsequent analysis. In addition, 496 normal brain samples with complete RNA-seq data (including tissues from different parts of the brain, such as the cerebrum and cerebellum) were used as a tumor-free cohort. Furthermore, considering that batch effects may exist between or within different databases, we used the R package “limma” of the “normalizeBetweenArrays” function to remove multiple batch effects (7).

### Clinical samples

This study was approved by the Ethics Committee of the Renmin Hospital of Wuhan University. Informed consent was obtained from all patients. A total of two normal brain tissue samples and four WHO 2/3 glioma samples (two WHO 2 samples and two WHO 3 samples) were collected between April 2018 and April 2022. None of the patients received chemotherapy or radiotherapy before surgery. The samples were used to verify the mRNA expression of survival-related independent risk factors in CARSs.

### Identification of differentially expressed genes

Data from the Genotype-Tissue Expression (GTEx) and TCGA databases were merged. Differentially expressed genes (DEGs) were identified from CRGs by the R package “limma” (8). The criteria were set as a false discovery rate (FDR) less than 0.05 and an absolute value of logFC greater than 0.7.

### Protein–protein interaction network analysis

A protein-protein interaction (PPI) network of ten CRGs was constructed with the STRING database. Nodes with an interaction confidence greater than 0.4 are shown. The correlation analysis of CRGs was performed by Bioladder.

### Construction of risk profiles associated with cuproptosis

The “survival” R package was used to perform univariate Cox regression to assess the prognostic value of CRGs in WHO 2/3 glioma (genes with P value< 0.05 were chosen for further study). The regression coefficients of gene expression were obtained using the least absolute shrinkage and selection operator (LASSO) regression algorithm (9). The formula for calculating the risk score was as follows:


$$Riskscore=\sum_{1}^{n}k_{n}\times A_{n}$$

where n is the number of prognosis-related genes, A_n_ is the expression level of prognosis-related genes, and k_n_ is the regression coefficient of prognosis-related genes.

### Principal component analysis

WHO 2/3 glioma samples were divided into high- and low-risk groups based on the median of the calculated risk scores. Principal component analysis (PCA) was used to investigate between-group differences.

### Prognostic analysis of the cuproptosis-associated risk signature

The prognostic value of CARSs in WHO 2/3 glioma was assessed by Kaplan-Meier survival analysis and Cox regression analysis in the training and validation cohorts (log-rank test P value< 0.05 was considered significant). In addition, a receiver operating characteristic (ROC) curve was derived for CARSs and other clinical risk factors to predict 1-year overall survival (OS) in WHO 2/3 glioma patients, and the area under the curve (AUC) was calculated from the ROC curve.

### Clinicopathological relevance of the cuproptosis-related risk signature

In the training and validation cohorts, patients were divided into high-risk and low-risk groups. We performed differential analysis of risk scores for five clinicopathological features, including WHO grade, IDH mutation, 1p19q codeletion, age, and gender, using the chi-square test. P values< 0.05 were considered significant.

### Tumor-infiltrating immune cells profiles

The abundance of immune cells in the low-risk group and high-risk group was estimated by the TIMER and Xcell databases, respectively. The Wilcoxon test investigated the association between tumor-infiltrating immune cells and risk scores in the TCGA and CGGA cohorts (p< 0.05 was considered significant).

### Single-sample gene sets enrichment analysis

Single-sample GSEA was used to calculate levels of tumor-infiltrating immune cells from WHO 2/3 glioma mRNA expression data. In addition, the enrichment of 29 immune-related markers was differentially analyzed in the low-risk and high-risk groups of the TCGA cohort and the CGGA cohort (p-value< 0.05 for significance). Furthermore, considering the importance of immune checkpoint molecules in cancer immunity, we subsequently analyzed their differential expression levels in low-risk and high-risk groups.

### Immunohistochemistry

Paraffin sections were prepared from WHO 2/3 glioma tissues acquired from patients. Afterward, they were deparaffinized and incubated with the primary antibody (Proteintech™) and then the secondary antibody (Proteintech™). Finally, the slides were stained, and images were captured with an Olympus BX40 microscope (Tokyo, Japan).

## Results

### Altered expression of cuproptosis-related genes in WHO 2/3 glioma

**Figure 1:**
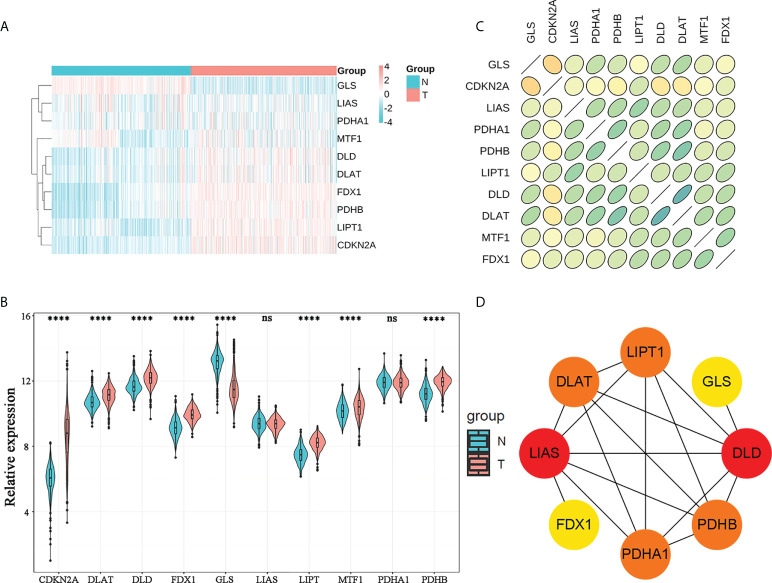
Genomic characterization of CRGs. **(A)** Heatmap of CRG expression in normal brain tissues and WHO 2/3 glioma tissues. **(B)** Violin plots of CRG expression in normal brain tissues and WHO 2/3 glioma tissues. **** P < 0.0001; ns, not significant. **(C)** Correlation plot of CRGs; green represents a positive correlation, yellow represents a negative correlation, and shades of color represent the degree of correlation. **(D)** PPI network of CRGs in the STRING database.

By differential analysis of ten CRGs in the training cohort, we screened eight DEGs: FDX1, PDHB, GLS, CDKN2A, DLAT, DLD, LIPT, and MTF1 (**Figures 1A, B**). The up- and downregulated CRGs and corresponding logFC values are shown in **Supplementary Table 1**. Furthermore, we found that there was a strong correlation between the expression of CRGs by Spearman correlation analysis (**Figure 1C**). The most correlated genes were DLAT and DLD. In addition, PPI network analysis also confirmed a strong expression correlation between CRGs (**Figure 1D**).

### Construction and verification of cuproptosis-associated risk signatures

**Figure 2:**
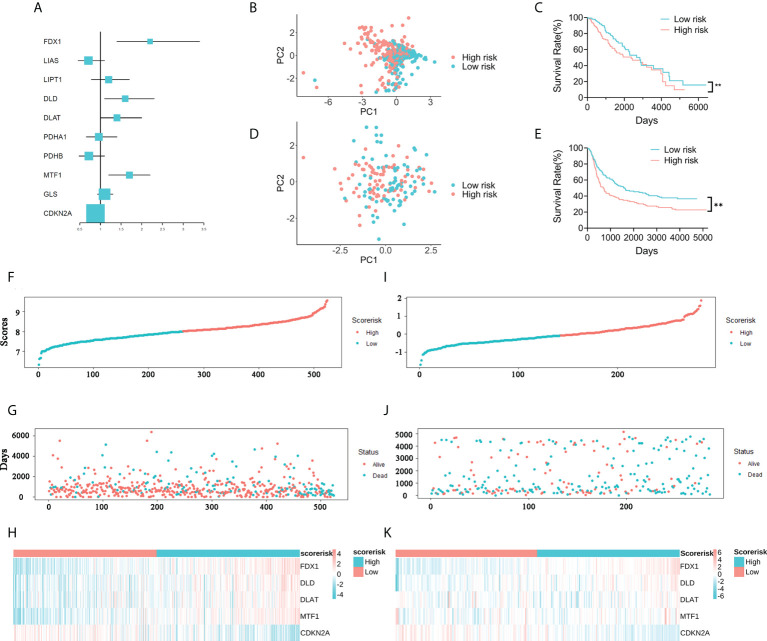
Construction of a five-gene CARS. **(A)** Forest plot for survival analysis of WHO 2/3 glioma patients using univariate Cox regression analysis in the TCGA cohort. **(B)** PCA of WHO 2/3 glioma samples in the TCGA cohort. **(C)** Overall survival analysis of WHO 2/3 glioma patient risk scores in the TCGA cohort. ** P < 0.01. **(D)** PCA of WHO 2/3 glioma patients in the CGGA cohort. **(E)** Survival analysis of WHO 2/3 glioma patients in the CGGA cohort. ** P < 0.01. **(F–H)** Distribution of risk scores, survival times and gene expression in WHO 2/3 glioma patients in the TCGA cohort. **(I-K)** Distribution of risk scores, survival times, and gene expression in WHO 2/3 glioma patients in the CGGA cohort.

A total of five prognosis-related genes (P< 0.05) were identified from the ten CRGs by univariate Cox regression analysis for further LASSO regression analysis (**Figure 2A**). The optimal prognostic model included five signature genes: FDX1, DLD, DLAT, MTF1, and CDKN2A. The five signature genes and their corresponding regression coefficients are listed in **Supplementary Table 2**. Then, the risk score of each patient was calculated based on the mRNA expression level of each risk gene and the corresponding coefficient. We noticed that the low-risk and high-risk groups could be effectively distinguished by PCA (**Figures 2B, D**). Kaplan-Meier survival analysis performed in the training and validation cohorts also showed that the low-risk group survived significantly longer than the high-risk group (**Figures 2C, E**). In addition, we plotted the distribution of risk gene expression, risk score, and survival status in the TCGA and CGGA cohorts (**Figures 2F–K**). Finally, we found by gene set enrichment analysis (GSEA) that cell adhesion, leukocyte transendothelial migration and toll like receptor signaling pathway were significantly activated in WHO 2/3 glioma tissues (**Supplementary Figure 1**). All the results demonstrated that the novel CARS-based risk scoring model could effectively predict the prognosis of WHO 2/3 glioma patients.

### Risk score may be an independent factor for overall survival prognosis in patients with WHO 2/3 glioma

**Figure 3:**
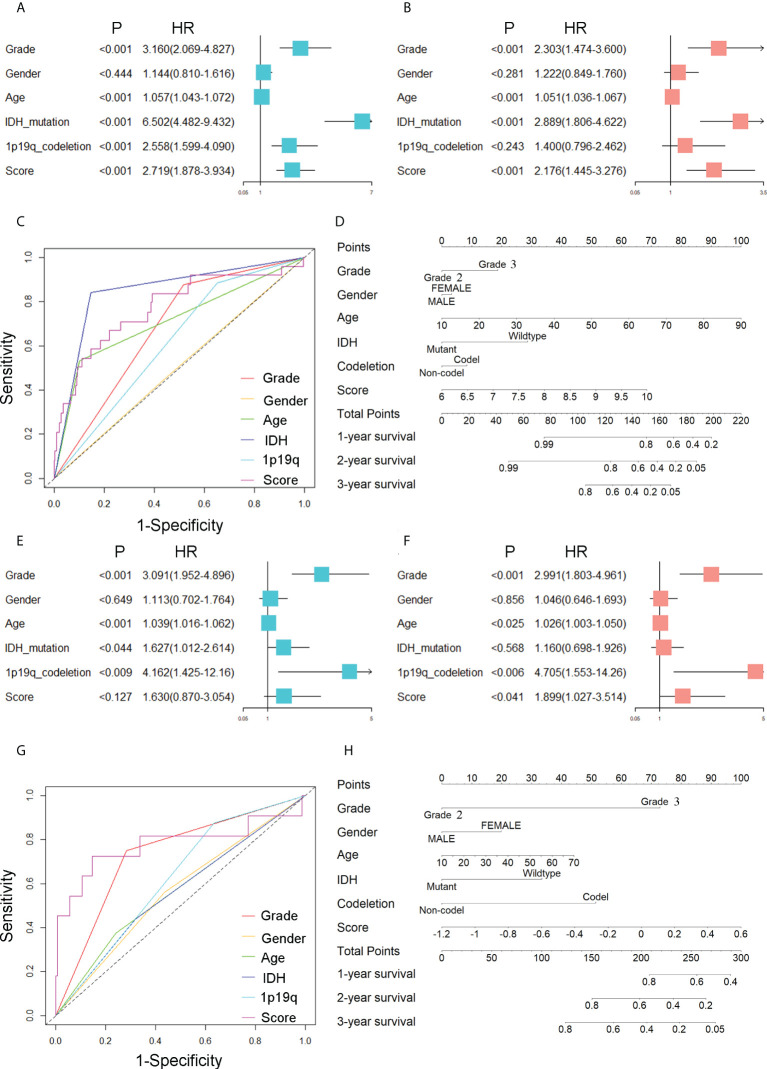
Prognostic value of CARSs. **(A)** Forest plot of univariate Cox regression analysis evaluating the prognostic value of risk scores and clinical factors in the TCGA cohort. **(B)** Forest plot of multivariate Cox regression analysis assessing the prognostic value of risk scores and clinical factors in the TCGA cohort. **(C)** ROC curves of risk scores and clinical factors in TCGA for predicting 1-year OS. **(D)** The nomogram based on the risk score and other clinical factors in the training cohort. **(E)** Forest plot of univariate Cox regression analysis evaluating the prognostic value of risk scores and clinical factors in the CGGA cohort. **(F)** Forest plot of multivariate Cox regression analysis assessing the prognostic value of risk scores and clinical factors in the CGGA cohort. **(G)** ROC curves of risk scores and clinical factors in CGGA for predicting 1-year OS. **(H)** The nomogram based on the risk score and other clinical factors in the validation cohort.

To demonstrate the significance of CARSs in independently predicting patient outcomes, we performed univariate and multivariate Cox regression analyses. In the TCGA cohort, the risk score was found to be a possible independent risk factor for predicting patient OS (HR: 2.176, p< 0.001) (**Figures 3A, B**). The CARS-based risk score had a larger area under the ROC compared to other clinical prognostic factors (WHO grade, age, 1p19q codeletion status and gender) (**Figure 3C**). The AUC for the risk score of 1-year OS in patients in the TCGA cohort was 0.774. The same conclusion was drawn in the CGGA cohort, with an HR of 1.899 for the risk score in multivariate Cox regression analysis (P< 0.05) (**Figures 3E, F**). The AUC of the risk score for 1-year OS in the CGGA cohort was 0.780 (**Figure 3G**). Further, to predict the 1-, 2-, and 3-year survival probabilities of WHO 2/3 glioma patients in the training and validation cohorts, we plotted nomograms based on the risk score and other clinical factors (**Figures 3D, H**).

### Relationship of cuproptosis-associated risk signatures with clinicopathological features

**Figure 4:**
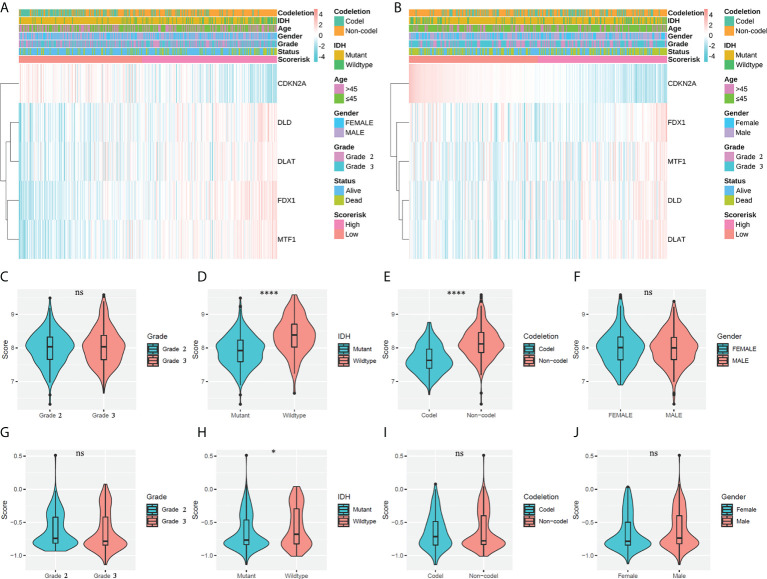
Associations between risk scores and clinicopathological factors. **(A)** Heatmap of the correlation between risk scores and clinicopathological features in the TCGA cohort. **(B)** Heatmap of the correlation between risk scores and clinicopathological features in the CGGA cohort. **(C–F)** Violin plot of the distribution of risk scores in patients stratified by WHO class, IDH status, 1p/19q codeletion status, and gender in the TCGA cohort. **** P < 0.0001; ns, not significant. **(G–J)** Violin plot of the distribution of risk scores in patients stratified by WHO class, IDH status, 1p/19q codeletion status, and gender in the CGGA cohort. * P < 0.05; ns, not significant.

A total of 452 cases in the training cohort and 345 cases in the validation cohort with sufficient data on age, gender, WHO class, IDH mutation status, and 1p19q codeletion status were screened to explore the relationship of CARSs with clinicopathological features. We found that CARS-based risk scores were significantly associated with IDH mutation status and 1p19q codeletion status in the TCGA cohort and with IDH mutation status in the CGGA cohort (**Figures 4A, B**). Specifically, in the TCGA cohort, samples with IDH wild-type or 1p19q noncodeletion had higher risk scores (**Figures 4D, E**); in the TCGA cohort, only samples with IDH wild-type had higher risk scores (**Figures 4H, I**). There was no significant association between the risk score and WHO class or gender in either the TCGA or CGGA cohorts (**Figures 4C, F, G, J**). Therefore, we speculated that the risk score was significantly associated with IDH mutations in WHO 2/3 glioma and possibly with 1p19q codeletion status.

### Validation of independent risk factors in cuproptosis-associated risk signatures

**Figure 5:**
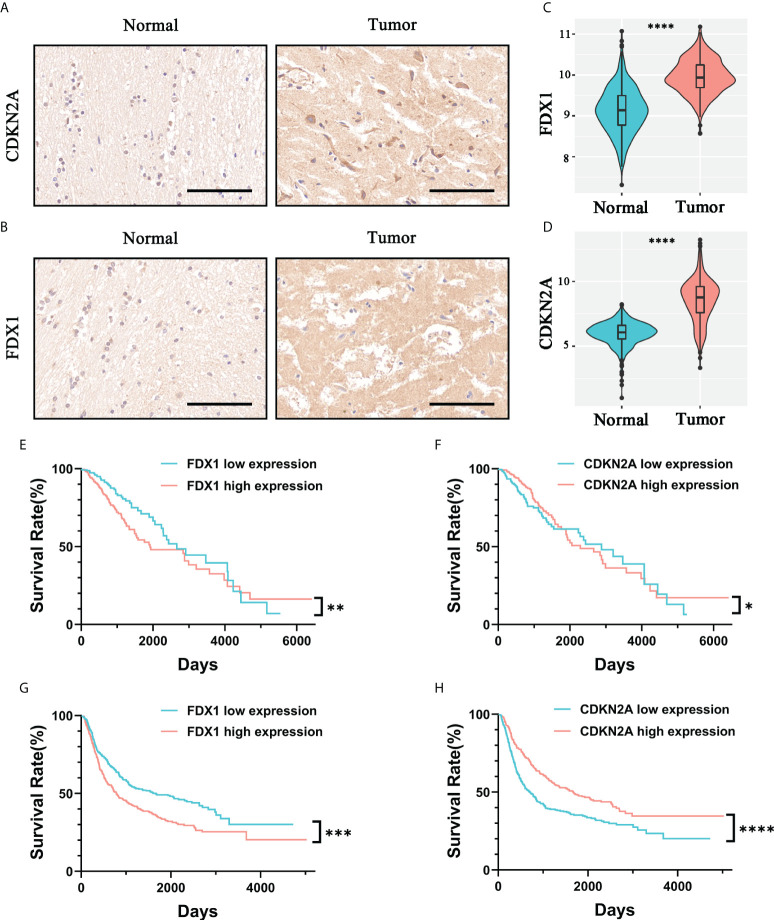
Validation of the prognostic value and expression of independent risk factors associated with prognosis in CARSs. The expression levels of CDKN2A **(A)** and FDX1 **(B)** in normal brain tissue and WHO 2/3 glioma tissue were investigated by immunohistochemistry. Scale bar, 100 μm. The expression levels of FDX1 **(C)** and CDKN2A **(D)** in normal brain tissue and WHO 2/3 glioma tissue were investigated in the TCGA cohort. **** P < 0.0001. **(E, F)** FDX1 and CDKN2A survival analysis of patients in the TCGA cohort. * P < 0.05; ** P < 0.01. **(G, H)** FDX1 and CDKN2A survival analysis of patients in the CGGA cohort. *** P < 0.001; **** P < 0.0001.

In the previous survival analysis, we identified two independent risk factors in CARSs by multivariate regression analysis: FDX1 and CDKN2A. We found that both FDX1 and CDKN2A were highly expressed in WHO 2/3 glioma tissue samples compared to normal brain tissue by immunohistochemistry (**Figures 5A, B**). In addition, by analyzing TCGA cohort mRNA expression data, we also found that FDX1 and CDKN2A were upregulated in WHO 2/3 glioma tissues (**Figures 5C, D**). Furthermore, we investigated the prognostic value of FDX1 and CDKN2A. Studies in the TCGA and CGGA cohorts showed that patients with low FDX1 expression had a longer prognosis (**Figures 5E, G**), while patients with high CDKN2A expression had a longer prognosis (**Figures 5F, H**).

### The immune infiltration characteristics of the risk score

**Figure 6:**
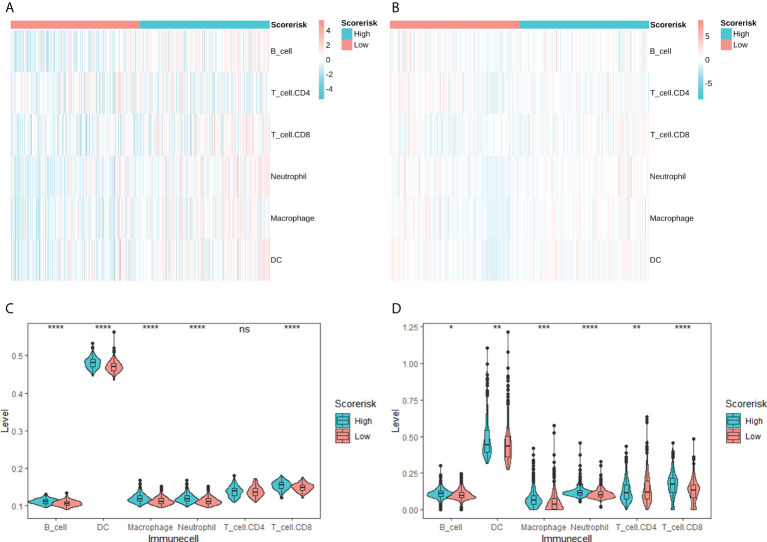
Correlation between CARS and tumor-infiltrating immune cells calculated by TIMER. Heatmap of tumor-infiltrating immune cells among low- and high-risk groups in TCGA **(A)** and CGGA **(B)** cohorts. Difference analysis of tumor-infiltrating immune cells, immune scores, and stromal scores in the TCGA cohort **(C)** and the CGGA cohort **(D)**. ns, not significant; * P < 0.05; ** P < 0.01; *** P < 0.001; **** P < 0.0001.

We studied the relative proportions of 6 types of immune cells based on the “TIMER” algorithm, and the results of differential analysis between low-risk and high-risk groups were shown in violin plots (**Figures 6A, B**). The abundance of B cells, dendritic cells, macrophages, neutrophils and CD8+ T cells in the high-risk group was significantly higher than that in the low-risk group (**Figures 6C, D**).

**Figure 7:**
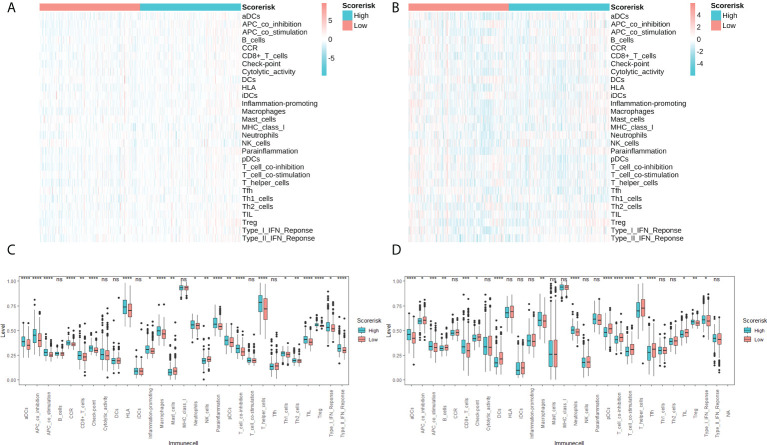
Single sample gene sets enrichment analysis (ssGSEA) of immune hallmarks. Heatmap of ssGSEA scores among low- and high-risk groups in TCGA **(A)** and CGGA **(B)** cohorts. Difference analysis of tumor-infiltrating immune cells, immune scores, and stromal scores in the TCGA cohort **(C)** and the CGGA cohort **(D)**. ns, not significant; * P < 0.05; ** P < 0.01; *** P < 0.001; **** P < 0.0001.

In addition, we quantitatively assessed the activity and abundance of immune cells based on the ssGSEA score. Samples with higher ssGSEA scores predicted a greater proportion of infiltrating immune cells and activity of immune-related pathways. For most immune cell types, samples in the high-risk group predicted higher ssGSEA scores, as shown in heatmaps (**Figures 7A, B**) and boxplots (**Figures 7C, D**). We found that patients with higher risk scores tended to have higher proportions of tumor-infiltrating immune cells (M2 macrophages and Tregs) and more active immune-related pathways. We also drawn the same conclusion with the “Xcell” algorithm, as shown in **Supplementary Figure 2**.

### Associations between risk scores and immune checkpoint molecules

**Figure 8:**
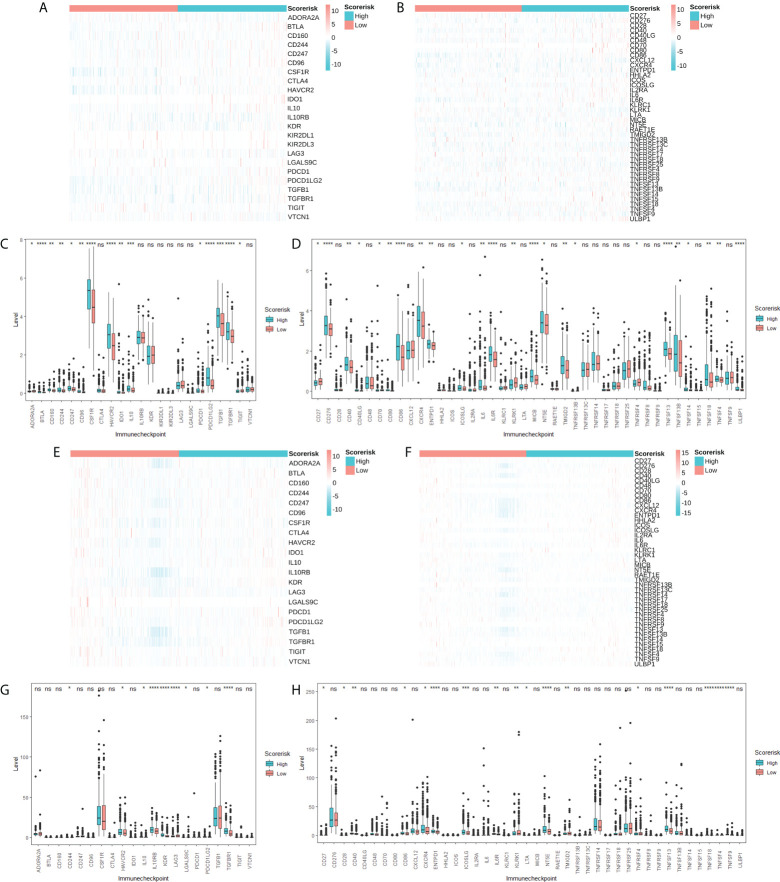
Association between CARS and immune checkpoint molecules. Heatmap **(A)** and difference analysis **(C)** of immunoinhibitor related molecules among low- and high-risk groups in TCGA cohorts. Heatmap **(B)** and difference analysis **(D)** of immunostimulator related molecules among low- and high-risk groups in TCGA cohorts. Heatmap **(E)** and difference analysis **(G)** of immunoinhibitor related molecules among low- and high-risk groups in CGGA cohorts. Heatmap **(F)** and difference analysis **(H)** of immunostimulator related molecules among low- and high-risk groups in CGGA cohorts. ns, not significant; * P < 0.05; ** P < 0.01; *** P < 0.001; **** P < 0.0001.

Considering the importance of immune checkpoint molecules in anticancer immunity, we investigated their expression levels in different risk groups. 5 immunosuppressor-related genes **(Figures 8A, C, E, G**) and 14 immune stimulator-related genes (**Figures 8B, D, F, H**) were differentially expressed in different risk groups. Therefore, the risk score can predict the expression level of immune checkpoint molecules and is regarded as a potential immunotherapy biomarker.

### Associations between risk scores and drug sensitivity

**Figure 9:**
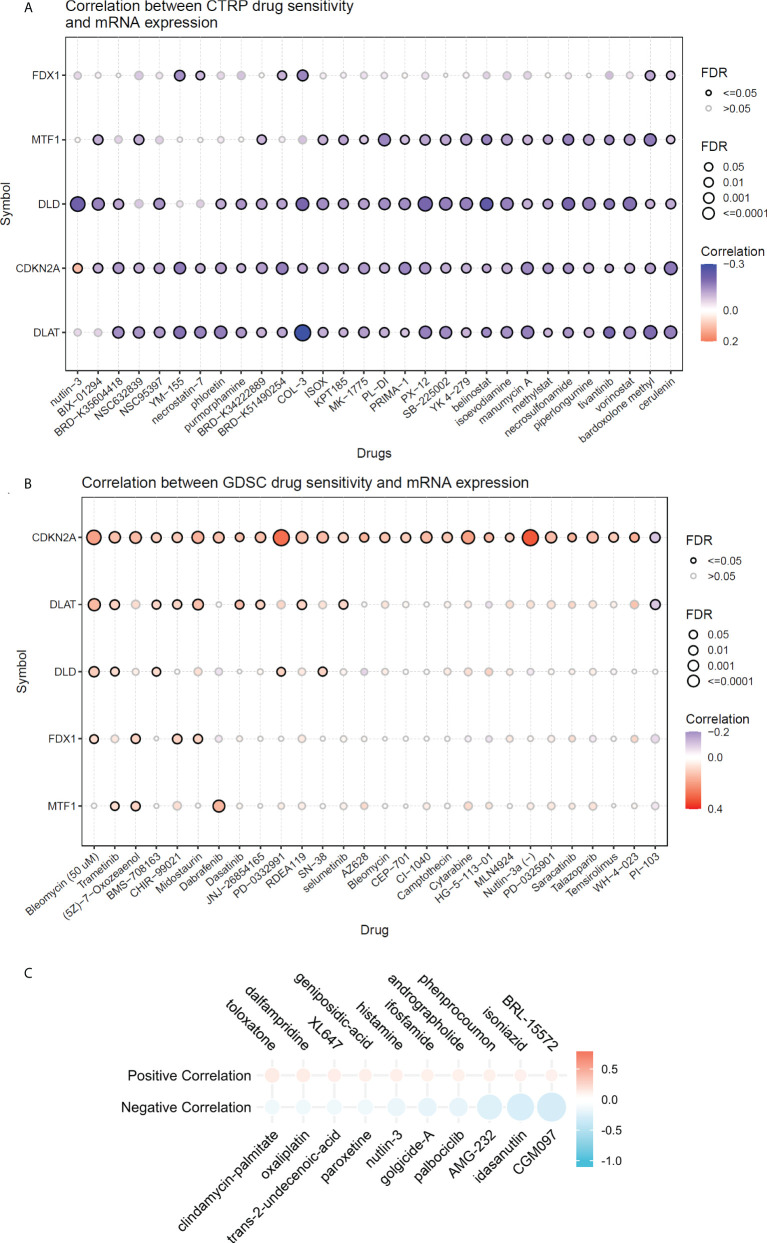
The relationship between risk scores and drug sensitivity. **(A)** The relationship between risk scores and drug sensitivity in CTRP database. **(B)** The relationship between risk scores and drug sensitivity in GDSC adtabase. **(C)** The relationship between risk scores and drug sensitivity in PRISM database.

Using the GDSC and CTRP databases, we summarized the correlation between risk scores and drug sensitivity in pan-cancer, and the 30 drugs with the strongest correlations have been listed (**Figures 9A, B**) (10). Further, we analyzed the association between risk scores and responsiveness of non-tumor drugs through the PRISM database (11) (**Figure 9C**). The results showed that six drugs, including toloxatone, dalfampridine, XL647, AMG-232, idasanutlin, and CGM097, had the strongest correlations with risk scores.

## Discussion

The TCA cycle is a common metabolic pathway for energy production in living organisms. Conventional wisdom holds that cancer cells bypass the TCA cycle and primarily utilize aerobic glycolysis. However, emerging evidence suggests that cancer cells with dysregulated expression of oncogenes and tumor suppressor genes rely heavily on the TCA cycle for energy production and synthesis of macromolecules (12). Inhibition of the TCA cycle is an important mechanism in tumorigenesis (13). In addition, studies have found active TCA cycles in glioma cells, which are characterized by feeding biosynthetic pathways (14). Therefore, we speculate that the TCA cycle is an important target for the treatment of glioma. Recently, cuproptosis was discovered as a novel programmed cell death pathway (4). Excess intracellular copper binds directly to the fatty acylated component of the TCA cycle, resulting in fatty acylated protein aggregation and subsequent loss of iron-sulfur cluster proteins, which in turn leads to proteotoxic stress and ultimately cell death. Ten CRGs were identified in this study. We started with the genome associated with cuproptosis and explored the expression and association of these genes in WHO 2/3 glioma. Then, five-gene CARSs were constructed and identified as novel prognostic biomarkers in WHO 2/3 glioma by internal and external validation.

In addition, we explored the relationship between risk scores and clinicopathological features. Furthermore, immunohistochemical staining of clinal WHO 2/3 glioma tissue confirmed the expression of independent risk genes associated with prognosis in CARSs; the expression of these genes was also verified with the data from the TCGA cohort.

Most of the genes associated with cuproptosis were differentially expressed in normal brain and WHO 2/3 glioma tissues. In this study, we constructed and validated CARSs of five genes. We found that risk signatures were more accurate in their prognostic predictive values than other clinically independent prognostic factors, which may provide effective individual mortality risk prediction and risk stratification for WHO 2/3 glioma patients. Risk scores did not show significant relationships with the WHO glioma grade, suggesting that risk characteristics may be unrelated to the degree of malignancy. However, IDH types and 1p/19q codeletion status in tumors can be distinguished by risk scores; IDH wild-type and 1p19q noncodeletion are often associated with resistance to conventional radiotherapy or chemotherapy in glioma patients and are important factors for poor prognosis in WHO 2/3 glioma (15, 16). Therefore, WHO 2/3 glioma patients with higher risk scores may be less sensitive to radiation or chemotherapy.

Due to the high heterogeneity of glioma, a single differentially expressed gene is usually not an effective biomarker for WHO 2/3 glioma patients, while a clinical prognostic model constructed by multiple DEGs can be better used for clinical applications and provide a reference for the treatment decisions of WHO 2/3 glioma patients (17).

Evasion of immune surveillance is one of the important mechanisms of tumorigenesis. It has been found that the induction of an immunosuppressive tumor microenvironment is mainly attributable to M2 macrophages (18). Further, excessive infiltration of M2 macrophages and Treg cells was associated with reduced overall survival. In this study, we found that higher risk scores predict higher M2-type macrophage and Treg cell infiltration, which may be a potential immunological explanation for poor prognosis. Further, we explored the relationship between risk scores and immune checkpoint molecules and chemotherapeutic drug resistance, which may provide references for subsequent immunotherapy and chemotherapy.

No previous study has investigated the correlation between CRGs and glioma development. Surprisingly, most CRGs were differentially expressed between tumor and normal tissues and were significantly associated with overall survival, suggesting a potential role of cuproptosis in the prognosis of WHO 2/3 glioma patients. There is evidence that copper ions can inhibit the activity of glioma cells, and the main mechanism is to promote the oxidation of proteins (19). However, our further study identified FDX1 and CDKN2A as independent prognostic factors, which was consistent with the existing findings (20). In addition to this, we also investigated the association of CARS with tumor immune infiltration. We found that higher risk scores corresponded to higher M2-type macrophage and Treg cell infiltration. This suggests that patients in the high-risk group may be potential beneficiaries of clinical tumor immunotherapy.

In this study, a novel prognostic biomarker based on CRGs constructed in WHO 2/3 glioma can effectively distinguish the IDH type and 1p/19q codeletion status and predict the effect of radiotherapy or chemotherapy on patients. However, it should be noted that the current research on glioma-related clinical prognostic models is in the development stage, and cuproptosis-based novel prognostic biomarkers still require further multicenter, prospective and randomized studies.

## Conclusion

To predict overall survival in WHO 2/3 glioma patients, we selected 5 CARSs from 10 CRGs. A clinical prognostic model based on CARSs was constructed and validated. The clinical prognostic model is expected to provide a reference for the treatment and prognosis evaluation of WHO 2/3 glioma patients.

## Data availability statement

The datasets presented in this study can be found in online repositories. The names of the repository/repositories and accession number(s) can be found in the article/**Supplementary Material**

## Ethics statement

The studies involving human participants were reviewed and approved by Ethics Committee of the Renmin Hospital of Wuhan University. The patients/participants provided their written informed consent to participate in this study.

## Author contributions

ZY: Conceptualization, Investigation, Writing - Original Draft; SZ: Methodology, Visualization, Writing - Review and Editing; JC: Investigation, Data Curation; LY: Investigation, Validation; LG: Methodology, Visualization; YXW: Data Curation, Visualization; ST: Resources, Investigation; QS: Validation, Data Curation; YW: Visualization, Resources; XX: Writing - Review and Editing, Supervision, Project administration, Funding acquisition; QC: Writing - Review and Editing, Supervision, Project administration, Funding acquisition. All authors contributed to the article and approved the submitted version.

## Funding

This work was supported by the National Natural Science Foundation of China (nos. 82001385, 81572489), the Natural Science Foundation of Hubei Province (nos. 2020CFB638) and the Science and Technology Application Project of Wuhan (nos. 2020020601012248).

## Conflict of interest

The authors declare that the research was conducted in the absence of any commercial or financial relationships that could be construed as a potential conflict of interest.

## Publisher’s note

All claims expressed in this article are solely those of the authors and do not necessarily represent those of their affiliated organizations, or those of the publisher, the editors and the reviewers. Any product that may be evaluated in this article, or claim that may be made by its manufacturer, is not guaranteed or endorsed by the publisher.

## Supplementary material

Supplementary Table 1 List of the up- and downregulated CRGs and their corresponding logFC values.

Supplementary Table 2 List of the five cuproptosis-associated risk signature genes and their corresponding regression coefficients.

Supplementary Figure 1 Gene Set Enrichment Analysis (GSEA) of CARS.

Supplementary Figure 2 Correlation between CARS and tumor-infiltrating immune cells calculated by Xcell. Heatmap of tumor-infiltrating immune cells among low- and high-risk groups in TCGA **(A)** and CGGA **(B)** cohorts. Difference analysis of tumor-infiltrating immune cells, immune scores, and stromal scores in the TCGA cohort **(C)** and the CGGA cohort **(D)**. ns, not significant; * P < 0.05; ** P < 0.01; *** P < 0.001; **** P < 0.0001.
