# Supplementary figures and images for: Development and validation of cuproptosis-associated prognostic signatures in WHO 2/3 glioma
Source: Front Oncol. 2022 Aug 18;12:967159. doi: 10.3389/fonc.2022.967159 (PMC9434124; doi:10.3389/fonc.2022.967159)

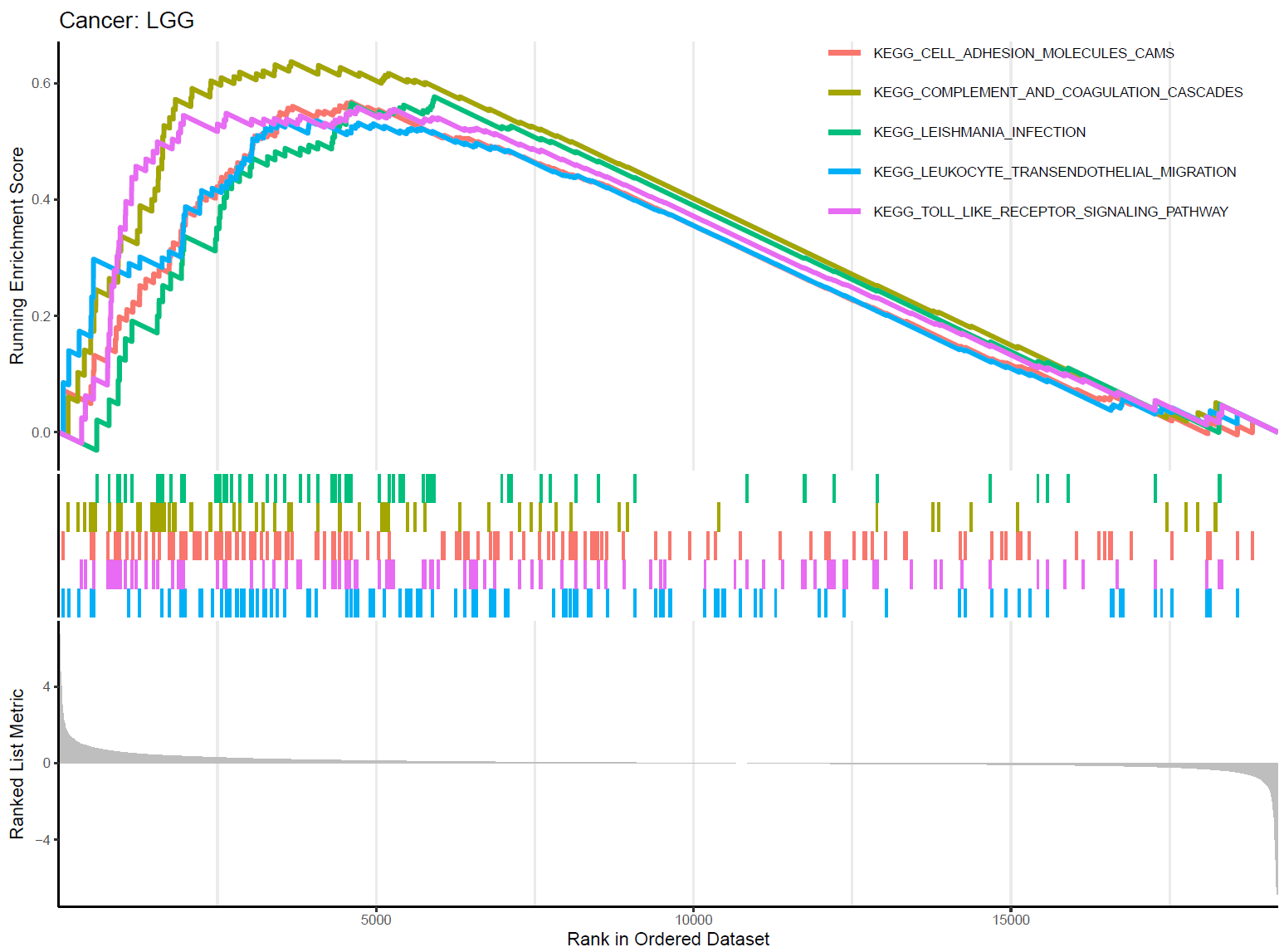

Supplement: Supplementary Table 1 — List of the up- and downregulated CRGs and their corresponding logFC values. [file Image_1.tif]

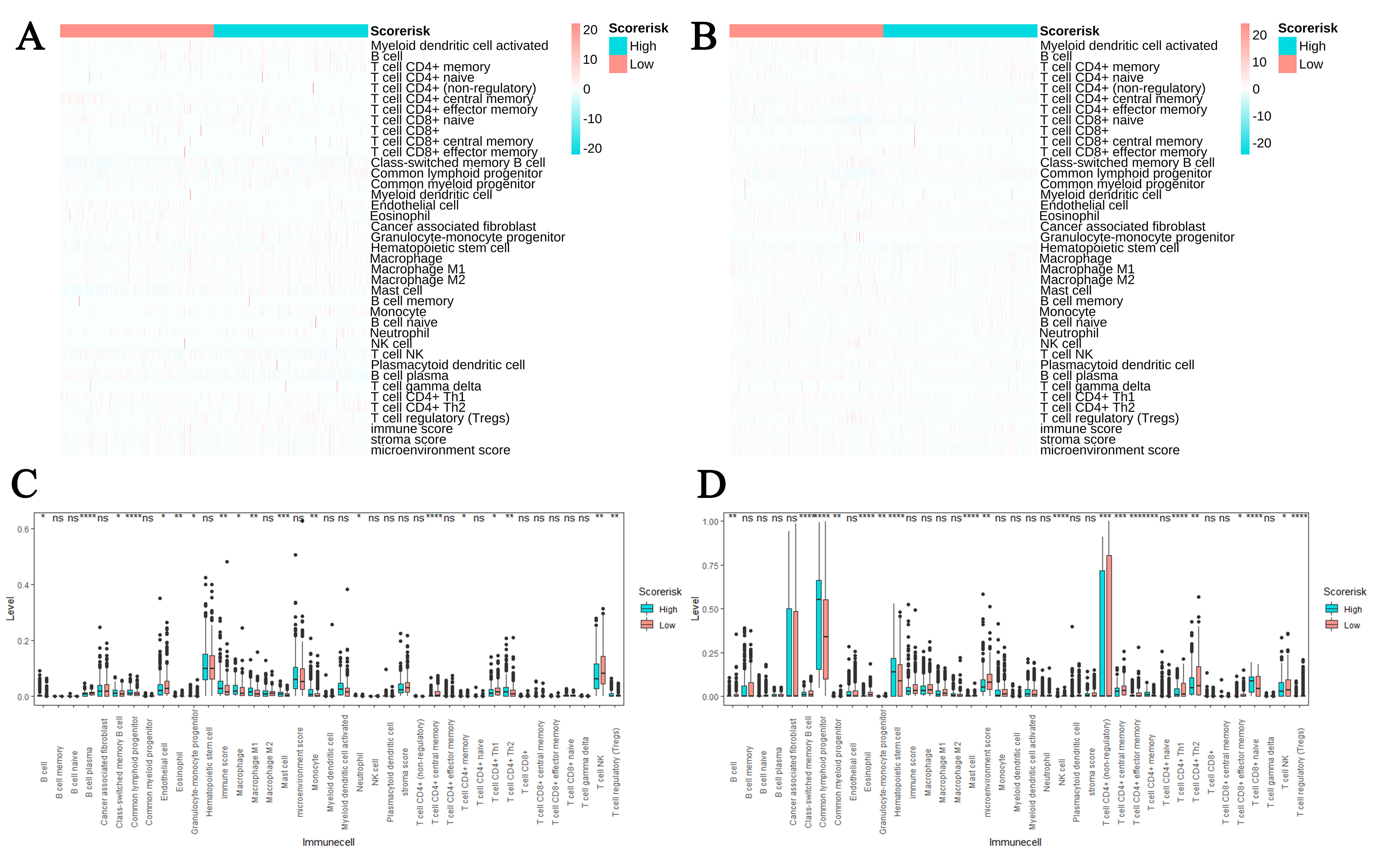

Supplement: Supplementary Table 2 — List of the five cuproptosis-associated risk signature genes and their corresponding regression coefficients. [file Image_2.tif]
