# Supplementary material for: Development and validation of cuproptosis-associated prognostic signatures in WHO 2/3 glioma
Source: Front Oncol. 2022 Aug 18;12:967159. doi: 10.3389/fonc.2022.967159 (PMC9434124; doi:10.3389/fonc.2022.967159)
Supplement: Supplementary Figure 1 — Gene Set Enrichment Analysis (GSEA) of CARS. [file Table_1.docx]

| Gene | logFC | P.Value |
| --- | --- | --- |
| CDKN2A | 2.643149 | 1.96E-149 |
| GLS | -1.55155 | 1.45E-143 |
| FDX1 | 0.817051 | 5.70E-121 |
| LIPT1 | 0.733389 | 8.15E-105 |
| PDHB | 0.717378 | 1.07E-100 |
| DLD | 0.493989 | 8.57E-50 |
| DLAT | 0.412726 | 1.77E-36 |
| MTF1 | 0.204379 | 5.01E-08 |
| LIAS | -0.05271 | 0.053364965 |
| PDHA1 | 0.003007 | 0.913712744 |
