# Supplementary material for: Development and validation of cuproptosis-associated prognostic signatures in WHO 2/3 glioma
Source: Front Oncol. 2022 Aug 18;12:967159. doi: 10.3389/fonc.2022.967159 (PMC9434124; doi:10.3389/fonc.2022.967159)
Supplement: Supplementary Figure 2 — Correlation between CARS and tumor-infiltrating immune cells calculated by Xcell. Heatmap of tumor-infiltrating immune cells among low- and high-risk groups in TCGA (A) and CGGA (B) cohorts. Difference analysis of tumor-infiltrating immune cells, immune scores, and stromal scores in the TCGA cohort (C) and the CGGA cohort (D). ns, not significant; * P < 0.05; ** P < 0.01; *** P < 0.001; **** P < 0.0001. [file Table_2.docx]

| Gene | coef |
| --- | --- |
| FDX1 | 0.8011 |
| DLD | 0.1055 |
| DLAT | -0.2998 |
| MTF1 | 0.3671 |
| CDKN2A | -0.2006 |
